# Stress and Skin: An Overview of Mind Body Therapies as a Treatment Strategy in Dermatology

**DOI:** 10.5826/dpc.1104a91

**Published:** 2021-10-01

**Authors:** Rachel Graubard, Ariadna Perez-Sanchez, Rajani Katta

**Affiliations:** 1 Baylor College of Medicine, Houston, TX, USA; 2 Department of Internal Medicine, University of Texas at San Antonio, USA; 3 McGovern Medical School at the University of Texas Houston, USA

**Keywords:** dermatology, stress, cognitive behavioral therapy, mind body therapies, biofeedback

## Abstract

Stress has multiple and wide-ranging physiologic and clinical impacts on skin disease. This has led to an interest in mind body therapies as potential adjunct treatments for skin disease. The stress response results in the activation of the endocrine, neurologic, and immune systems, with a resulting cascade of impacts, that are both systemic and cutaneous. The 2 main arms of the stress response are the sympathetic nervous system and the hypothalamic-pituitary-adrenal axis. The resultant release of cortisol, catecholamines, and neuropeptides has multiple effects. Clinically, these have been shown to increase skin inflammation, increase itching, impair skin barrier function, impair wound healing, and suppress immunity.

Mind body therapies are those that focus on the interaction between the mind and the body, with the goal to influence physical function and impact health. These have been shown to ameliorate some of the harmful physiologic changes attributed to stress or to reduce harmful behaviors. In some cases, such as with biofeedback, they may also result in beneficial physiologic changes.

Treatments such as meditation, biofeedback, hypnosis, guided imagery, and others have been evaluated in the treatment of skin disease and have shown some benefits. Although randomized controlled trials are limited, these interventions have shown beneficial effects on itching, psychosocial outcomes, and even skin severity. These interventions have been evaluated in diseases such as atopic dermatitis, psoriasis, trichotillomania, and others. Given the potential benefits, improvements in psychosocial outcomes, and a low risk profile, referral to qualified practitioners or multidisciplinary clinics should be considered for some patients.

## Introduction

Research has documented that stress has wide-ranging physiologic and clinical impacts on skin disease [1]. This has led to an interest in mind body therapies (MBTs) as potential adjunct therapies in the treatment of skin disease.

Stress has been defined by the National Cancer Institute as the body‘s response to physical, mental, or emotional pressure [2]. This stress response may include both conscious and unconscious changes. Stress can also refer to an emotional response. Cohen et al write that “the psychological stress response is composed of negative cognitive and emotional states and occurs when demands imposed by events exceed a person’s ability to cope”[3].

A number of potential events or circumstances may produce a stress response, these are known as stressors. Stress may be either acute or chronic and is determined by an individual’s perception and response to the stressor, rather than inciting circumstances. In dermatology, stress may affect skin in a variety of ways, including physiologic changes or increases in behaviors (such as scratching) that ultimately worsen skin disease. In addition, stress may arise from a skin disorder itself, resulting in a self-perpetuating cycle.

A number of MBTs have been evaluated for their potential impact on stress and skin disease [4]. This review will describe the potential mechanism of action and evidence for utility in the treatment of skin disease for several Mind Body Medicine (MBM) modalities, including biofeedback, behavioral and cognitive behavioral therapy, meditation, hypnosis, and relaxation therapies.

### Physiologic Impact of Stress on the Skin

The skin acts not only as a physical barrier to the external environment, it may outwardly express the manifestations of internal processes. Through its network of mechanical and chemical receptors, nerves, musculature, and vasculature, the skin interacts closely with the Central Nervous System (CNS) to respond to both physical and emotional stimuli. The skin is particularly sensitive to the effects of stress, either as the primary detector or the secondary receiver of the central stress response.

The stress response results in activation of the endocrine, neurologic, and immune systems, with a resulting cascade of events[5]. The 2 main arms of the stress response are the Sympathetic Nervous System (SNS) and the Hypothalamic-Pituitary-Adrenal (HPA) axis [6]. Activation of the SNS results in release of the catecholamines norepinephrine and epinephrine. This response is also known as the “fight or flight response” and impacts multiple organ systems. At the level of the HPA axis, stress triggers the hypothalamus to produce corticotropin-releasing hormone (CRH), which induces the release of adrenocorticotropic hormone (ACTH) from the pituitary gland, culminating with the release of cortisol from the adrenal glands.

The release of cortisol, catecholamines, and neuropeptides has multiple systemic and cutaneous effects. In a meta-analysis investigating the relationship between psychological stress and the immune system in human subjects, chronic stressors were associated with suppression of both cellular and humoral measures [7]. In human subjects, psychological stress was associated with an increased risk of acute respiratory illness following exposure to viruses, with the dose of stress correlated to risk of infection [3].

The release of neurohormones, neuropeptides, and neurotransmitters from both arms of the stress response impacts the skin [8]. Catecholamines may directly impact glands, blood vessels, and smooth muscles, while CRH and cortisol have multiple, wide-ranging effects. In addition, the skin is not only a target for mediators; it is also an active participant, specifically via a local HPA axis, peripheral nerves, and skin cells including mast cells, immune cells, and keratinocytes [9].

Taken together, mediators released systemically or locally have been shown to upregulate the production of other mediators, such as histamine and serotonin, increase neurogenic inflammation, increase the activation of sensory innervation, and decrease the itch-sensing threshold in itch-specific receptors [10]. These mediators may ultimately increase skin inflammation, increase itching, impair skin barrier function, impair wound healing, and suppress immunity [5,8].

### Clinical Impact on Skin Disease

Jafferany categorized the skin conditions affected by stress including psychophysiologic disorders, which are defined as “skin diseases that are precipitated or exacerbated by psychological stress”. These include acne, alopecia areata, atopic dermatitis, psoriasis, rosacea, chronic spontaneous urticaria, and others [11].

Clinically, this has been documented in multiple human studies. 2 separate prospective cohort studies reported a significant association between increased stress levels and acne severity [12, 13]. In a review of the effects of psychological stress on wound healing, the majority of studies found that stress was associated with impaired healing or dysregulation of a biomarker associated with wound healing [14].

Another category is that of psychological disorders with dermatologic symptoms, such as obsessive-compulsive disorder or anxiety resulting in acne excoriee, trichotillomania, and other disorders. In addition, dermatologic disorders may have associated psychological symptoms, such as anxiety, depression, and other mood disorders related to the presence of chronic eczema, psoriasis, vitiligo and other skin disorders [11].

### Mind Body Therapies

Mind-body therapies (MBTs) have been defined as therapies that “focus on the interaction between the mind and the body, with the intent to use the mind to influence physical functions and directly affect health” [15]. These therapies may ameliorate some of the harmful physiologic changes attributed to stress. They may also help reduce harmful behaviors. In some cases, such as biofeedback, they may result in beneficial physiologic changes.

MBTs include meditation, mindfulness-based stress reduction (MBSR), hypnotherapy, biofeedback, guided imagery, and others [15]. These are considered low risk and relatively low cost, and provide mental and physical health benefits [16].

Research in the use of these therapies can be challenging, as interventions may overlap or may be combined. Although randomized controlled trials are limited, the use of MBTs has shown overall benefit for those with skin disorders. In a meta-analysis of psychological interventions for adults with skin disorders, it was noted that these interventions overall had a medium-sized effect on itch/scratch (8 studies) and psychosocial outcomes (17 studies) [17]. A small to medium effect on skin severity was noted (17 studies). When analyzed further, a medium effect was noted for adults with psoriasis and atopic dermatitis as compared to controls [17].

It is important to stress that these therapies are to be considered adjuncts to, and not replacements for, standard medical therapy. As Fried and Hussain wrote, these are “analogous to corticosteroid-sparing therapy. Incorporating these techniques into conventional treatments has demonstrated efficacy in decreasing the amount of medication and ultraviolet exposure necessary to improve symptoms in psoriasis and eczema” [18]. Although these are distinct therapies, researchers have noted similarities of underlying principles, such as with hypnosis and meditation. Shenefelt stated that both “use natural trance states” [19]. Trance is described as a shift of brain waves that can occur naturally, such as when deeply absorbed in an activity or during repetitive strong activity. During trance states, differences are seen in regional cerebral blood flow and EEG patterns as compared with the typical waking state. Similarly, relaxation therapies encompass a number of distinct techniques, which have in common the ability to reduce arousal of the SNS.

It is also recognized that, although discussed separately, these techniques may have overlapping features. For example, “guided imagery and clinical hypnosis have significant overlap, and many studies combine these modalities.” [15].

A number of researchers have developed or studied interventions that combine these methods. Jon Kabat-Zinn developed the mindfulness-based stress reduction program (MBSR), which consists of an 8-week course and home practice. The core of the program centers on meditation practice, guided body scan, and yoga exercises [20].

### Biofeedback

Biofeedback involves the use of a device to measure a physiologic parameter, and then using visual, auditory, or tactile cues to make the patient aware. As a patient observes these measurements, they can practice controlling the parameter [21]. The most common types of biofeedback provide information on the ANS, and may include peripheral temperature measurements (hands or fingers), heart rate variability (HVR), or galvanic skin resistance (sweat gland activity) [15]. It has been shown that even children may successfully alter these parameters. In an early study, 48 children (5–15 years old) were able to raise and lower their index finger temperature with self-hypnosis and/or biofeedback [22].

Clinically, 11 of 14 adults with hyperhidrosis showed clinical improvement 6 weeks after completing biofeedback treatment [23]. In a study of patients with dyshidrotic eczema, 33 patients were trained to successfully decrease skin conductance and showed clinical improvement and decreased anxiety [24].

### Behavioral Therapy and Cognitive Behavioral Therapy

Habit reversal (HR) is one type of behavioral therapy, used to help reduce habits such as scratching [25]. HR is described as being easy to learn and “essentially a self-directed approach”, although instruction is required [26].

In HR, the main components are awareness (making the patient aware of their own behaviors) and competing response (teaching patients to practice alternate strategies in place of the target behavior). Other aspects of this therapy include stimulus control, relaxation training, and recruiting social support [26].

HR has shown success in the treatment of atopic dermatitis as well as repetitive behaviors such as skin picking and hair pulling [27]. 3 randomized controlled trials of HR in atopic dermatitis noted a significant reduction in severity and scratching, although the number of subjects was small and follow-up periods were brief [28]. In a review of studies of psychological interventions, HR was associated with the greatest impact on outcomes for subjects with skin disorders [17].

In 1 trial, use of HR in conjunction with standard therapy led to clinically significant reduction in eczema severity and improved quality of life for up to 1 year [29]. In another, use of HR with potent topical steroids for 3 weeks resulted in significantly improved skin severity in atopic dermatitis as compared to controls [30].

Cognitive behavioral therapy (CBT) adds a focus on thought patterns and has shown success in several skin disorders. In CBT, the goal is to alter dysfunctional habits “by interrupting and altering dysfunctional thought patterns (cognitions) or actions (behaviors) that damage the skin or interfere with dermatologic therapy” [31].

Both CBT and HR have shown promising results in the treatment of trichotillomania [32]. In psoriasis, patients undergoing an adjunctive CBT program exhibited greater improvement in symptoms’ clinical severity, reduced stress levels, anxiety, and depression compared to patients receiving standard pharmacological treatment alone [33]. In atopic dermatitis, adjunct treatments including CBT or education with CBT led to significantly greater improvements in skin condition and reduction in topical steroid use, as compared to standard medical therapy at 1-year follow-up [34].

### Meditation

Meditation has been defined as the practice of “intentional attention training”, and may be achieved via different approaches [15]. Meditation practices have been broadly divided into concentrative meditation and mindfulness meditation [35]. In the former, patients are asked to focus their attention on an object, image, or word. In the latter, patients are asked to be mindful of the present moment, with awareness of stimuli but without judgment.

In a systematic review of complementary therapies for psoriasis, meditation and guided imagery therapies showed modest efficacy in 3 single-blind randomized controlled trials [36]. Kabat-Zin evaluated the effects of a MBSR program in patients with moderate to severe psoriasis, in which some patients receive phototherapy alone and others participated in the program by listening to an audio tape during phototherapy for 13 weeks. Those in the MBSR group had significantly faster clearing of psoriatic lesions. Although promising, post intervention lasted only 1 week, and did have a high dropout rate [37]. In another study, subjects who completed an 8-week mindfulness program in conjunction with their usual therapy reported a significant improvement in both self-reported psoriasis severity and quality of life as compared to controls receiving only usual therapy [38].

### Hypnosis

Hypnosis has been defined as “the intentional induction, deepening, maintenance, and termination of the trance state for a specific purpose”[39]. Hypnosis creates an altered state of consciousness that renders the mind vulnerable to the power of suggestion. It is believed that through accessing the subconscious mind, an individual’s emotions, behaviors, and physiological responses can be influenced. While it may represent a useful adjunct therapy, it requires access to trained practitioners as well as careful subject selection, as some subjects exhibit greater hypnotic susceptibility than others [40].

Hypnosis has been employed in dermatology to reduce pain and pruritus from skin disorders, to reduce procedure-related anxiety, to reduce harmful behaviors, and as an aid for healing skin disease [40, 41].

A trial conducted on atopic dermatitis patients that were refractory to traditional therapies, reported statistically significant improvements in scratching, discomfort, and sleep disturbances, following hypnotherapy with direct suggestions of scratching cessation and skin comfort. In addition, corticosteroid use decreased by 60% at 16 weeks [42]. In a small randomized controlled trial, adults with psoriasis who were highly hypnotizable demonstrated significantly greater improvement in skin severity with hypnotherapy (in conjunction with conventional treatment) as compared to moderately hypnotizable subjects [43].

Hypnosis has also been used to help control harmful habits, such as scratching or picking [39]. 3 pediatric patients with trichotillomania responded well, and at 16 months none showed recurrence [44].

Hypnosis may also be used as adjunct therapy in the treatment of verruca vulgaris. In 1 interesting trial, 17 patients with bilateral warts were hypnotized and given the suggestion that the warts would improve on one side only. In 3 months, 53% of the experimental group showed resolution on the treated side alone, as compared to no improvement, in a control group receiving no therapy [45]. In another trial, subjects who received hypnotic suggestion showed greater remission of warts than a placebo light treatment or a control group who received no treatment [46].

### Relaxation Therapies That Reduce Arousal: Guided Imagery, Progressive Muscle Relaxation, and Others

Activation of the SNS at times of stress, leads to a state of physiologic arousal, with increases in heart rate, blood pressure, and other parameters. By contrast, the relaxation response is considered the “physiologic and psychologic opposite of the... stress response” [16]. The relaxation response has been described as a physiological state characterized by decreased arousal of the SNS [47], evidenced by a decrease in heart rate and respiratory rate along with an increase in certain brain waves and skin resistance[47].

Dr. Herbert Benson, a pioneer in its clinical applications, describes the relaxation response as a state rather than a specific technique, that can be elicited by multiple methods [47]. Several studies have used techniques such as meditation, progressive muscle relaxation, rhythmic breathing, imagery, autogenic training, and others to elicit the response [47]. “Physiologically, the techniques are effective in reducing sympathetic reactivity and enhancing parasympathetic activity” [18]. It is believed that breathing techniques that focus on a low respiration rate with long exhalations may be a key feature in activating the parasympathetic nervous system, possibly through stimulation of the vagus nerve [48].

One review evaluated the results of 37 studies of interventions that produced a relaxation response. Overall, these may be effective in reducing hypertension, insomnia, anxiety, pain, and medication use across multiple populations [49].

A number of relaxation techniques have been studied. Also known as “arousal reduction” techniques, these are frequently used in conjunction with other therapies. Some of the modalities discussed earlier may also independently result in a relaxation response.

Ersser et al describe several specific relaxation techniques [25]. Progressive muscular relaxation involves tensing different muscles in the body and then releasing the tension, enabling the individual to consciously learn how to release tension. Guided imagery is the use of relaxing or calming imagery, invoking all the senses, to help induce a similar feeling in the body [15]. In autogenic training, patients focus on specific parts of their body and use autosuggestions such as “skin calm and pleasantly cool” [34].

A randomized controlled trial studied the effects of progressive muscle relaxation in atopic dermatitis, and found significant decreases in pruritus and loss of sleep as compared to controls after 1 month [50]. In another randomized controlled trial, listening to 20 minutes of guided relaxation (either before or after skin wounding) led to improved skin barrier recovery as compared with a control group [51]. In a small study, children with atopic dermatitis (ages 5–15 years) were treated with hypnotherapy or a biofeedback device based on galvanic skin resistance (as a relaxation technique), and showed significant reduction in severity of surface damage and lichenification as compared with controls [52].

## Conclusion

MBTs may be considered as adjunct therapy in the treatment of several dermatologic conditions, including atopic dermatitis, psoriasis, self-induced skin conditions, and others. Other indications include during dermatologic procedures [35].

Further research is needed to confirm beneficial effects, to determine patient selection, and to delineate mechanisms of action. However, many of these therapies are low risk and referral to experienced practitioners or multidisciplinary clinics should be considered [4].
